# The impact of wearing a heart rate monitoring wristband on museum visitors’ memory and emotions: a randomized controlled trial

**DOI:** 10.1186/s41235-025-00630-9

**Published:** 2025-05-13

**Authors:** Nicola Vasta, Margherita Andrao, Barbara Treccani, Denis Isaia, Claudio Mulatti

**Affiliations:** 1 Department of Psychology and Cognitive Science, University of Trento, Corso Bettini 31, 38068 Rovereto, TN Italy; 2 Human Systems Lab, Department of Kinesiology, University of Windsor, Windsor, ON Canada; 3 Fondazione Bruno Kessler (FBK), Trento, Italy; 4 Museo di Arte Moderna e Contemporanea di Trento e Rovereto (MART), Rovereto, Italy

**Keywords:** Improved memory recall, Aesthetic emotion, Physiological measure, Museum, Art exhibition, Fake feedback

## Abstract

Advances in technology have enabled museum curators to employ equipment that can measure visitors’ physiological responses, offering a means to monitor these responses, while, at the same time, potentially engaging visitors. However, it is unclear whether these devices genuinely promote a positive experience or, conversely, are perceived as intrusive monitoring tools. Following traditional theories linking physiological responses, emotions and memory, we tested whether wearing a heart rate monitoring wristband during a temporary art exhibition could affect visitors’ emotions and if emotional changes due to this manipulation could, in turn, affect the long-term memory of the artworks. Our findings show that using such a device heightened pleasant emotions experienced by visitors and improved their memory of the exhibit. These effects were still present even after six days from the visit. Moreover, we found that providing fake feedback concerning the emotions experienced in a specific room increased visitors’ memory of artworks within that room. Our results are encouraging regarding the use of these technologies in museum exhibitions and bring evidence that they can enhance visitors' experiences, regardless of their accuracy.

## Significance statement

Visiting museums can promote well-being and evoke pleasant emotions in many individuals. Despite this, museum attendance remains low, highlighting the need to test novel technologies that can attract visitors by improving their emotional experiences and memories during their visits. According to traditional psychological theories, shifts in emotions are typically associated with changes in physiological indices; for instance, fear and anger are usually associated with an increased heart rate. Previous studies have also shown that experiences accompanied by richer and stronger emotional and physiological changes are better remembered. This study aims to leverage this knowledge to investigate the possibility of enhancing visitors' experiences of an art exhibition and their memory of the artworks through devices that measure physiological responses.

In this study, participants attending a temporary art exhibition were asked to fill an emotional questionnaire and perform a memory task. Half of the participants wore a physiological-measurement wristband during the visit, while the other half did not. All participants received fake feedback concerning their physiological/emotional experience in one specific room of the exhibition. The results not only revealed that the feedback improved participants’ memory of the artworks in the feedback room, but also showed that participants wearing the wristband both reported experiencing more pleasant emotions and demonstrated enhanced memory of the whole exhibition, even after six days or more. Our findings are encouraging regarding the use of these technologies in museum exhibitions and bring evidence that they enhance visitors' experiences, even without providing accurate feedback.

## Introduction

Research has shown that museums have the potential to relieve stress and replenish the attentional resources depleted by everyday life in many individuals (Kaplan et al., 1993; Packer, 2008). Despite this, in recent years, museum attendance has declined, with two out of three museums in the USA failing to return to pre-pandemic attendance levels (American Alliance of Museums, 2023). One way for museum curators to attract visitors is by emotionally engaging them and ensuring they remember the experience positively. However, making an exhibition attractive, enjoyable and memorable can prove to be a rather cumbersome process. Interactive installations, for example, are usually appreciated by both young (Haywood & Cairns, 2006) and adult visitors (Ciolfi & McLoughlin, 2012), but require the museum to invest considerable money and sometimes disrupt the original structure of the exhibit. In this study, we investigated whether the mere belief that physiological variables associated with emotions were being monitored during an exhibition visit, along with receiving feedback about them, can impact visitors' overall experience and memory of the visit.

With the development of modern equipment for measuring physiological indices (i.e. skin conductance, heart rate, etc.), general interest in the possibility of monitoring one’s physiological experience during a museum tour has grown (see Tschacher, et al., 2012, for a pioneering study on this topic). For instance, when visiting Google's art installation “A Space for Being'' at the 2019 Milan Design Week, visitors were invited to wear a wristband that monitored heart rate, breath rate, and skin temperature (cf., Magsamen & Ross, 2023). After taking a brief tour of some evocative interior spaces, visitors received feedback on their physiological reactions during the visit. In this way, visitors could compare information about their physiological activation with their emotional experience, while museum curators could be provided with insights into the specific locations where visitors exhibited different physiological reactions.

Physiological responses are a critical component of emotions. Typically, in response to information with emotional meaning (e.g. a particularly striking piece of museum art), a widespread activation of both the visceral and somatic systems occurs. This results in physiological responses that may include variations in the heart rate and blood pressure, sweating, and other measurable bodily changes, all of which can be captured using appropriate devices.

There is evidence that specific emotions, such as happiness, sadness, fear, and disgust, can be distinguished by their physiological patterns (Ekman, et al., 1983; Ekman & Davidson, 1994; Levenson, et al., 1990), or, at least, that different physiological patterns characterize critical dimensions of emotions. Emotions are often described in terms of two dimensions: valence and arousal. Valence refers to whether the emotion is perceived as negative or positive, while arousal reflects its intensity (e.g. Barrett & Russell, 1999; Citron et al., 2014). Physiological responses linked to the activation of the visceral system (e.g. electrodermal activity, heart rate) are more reliably associated with changes in emotional arousal than in valence (cf., e.g. Bruin et al., 2024). This is consistent with theoretical approaches suggesting that valence and arousal are independent of one another and that (visceral) physiological activation simply reflects arousal (i.e. the intensity or strength of the emotion), while being undifferentiated with respect to the valence of the emotion. This undifferentiated physiological activation would then be interpreted (or appraised)—that is, a cognitive label would be applied to it—which determines its valence (positive or negative (e.g. Kissin, 2012; Rickard, 2004; Schachter & Singer, 1962). Other theoretical models propose instead some sort of interrelationship between the two dimensions, or, even when assuming their independence, suggest that different valences would also be differentiated by distinct physiological patterns,^1^ even though these patterns may be less easily distinguishable based on the currently available physiological measures compared to those characterizing different levels of arousal (cf., e.g. Fruet et al., 2024; Britton et al., 2006; see also Kuppens et al., 2013, for a discussion of the relation between valence and arousal).

Regardless of which aspects of emotions are reliably reflected by the psychophysiological responses captured by the currently available devices, there is agreement that changes in emotional states (in terms of valence and/or arousal) are reflected in measurable changes in psychophysiological responses. This can be leveraged to enhance the visitor experience of an exhibition: curators could leverage physiological data to identify which parts of the installation evoked emotional changes. However, despite the promising potential of this methodology, little is known about how visitors actually perceive and are affected by information about their physiological activation, or whether such information can positively influence their experience of the exhibition.

Interestingly, researchers have found that the more people are aware of their internal physiological activities, the more intense the emotions they experience (Pollatos & Schandry, 2008; Wiens, et al., 2000). Generally, the intensity and saliency of emotions increase by raising awareness of them. The awareness of one’s own emotions can be enhanced by providing appropriate and reliable feedback about physiological responses related to these emotions (cf., e.g. Carver & Scheier, 1978; Scheier & Carver, 1977). However, it might also be increased simply by letting people know that these responses are recorded, and such knowledge might influence the conscious processing of one’s emotional states: the mere knowledge that one's emotions are being measured can enhance the awareness of the experienced emotion as self-directed attention cause increased awareness of internal states (Scheier et al., 1979), potentially altering either the nature or intensity of the emotion itself. For instance, Kassam and Mendes (2013) observed different physiological reactions during a math task between participants who were asked to report their current emotions (thereby being primed to be aware of them) and participants who were not. Moreover, in studies designed to manipulate or measure affective states, there is evidence that participants' emotional and psychophysiological responses (e.g. heart rate) can be influenced simply by the awareness that these responses are being observed or recorded (cf., e.g. Larkin, et al., 1998; Liu et al., 2024). These observations are particularly interesting in the context of using devices to record psychophysiological responses in real-world settings, as physiological measurements obtained in uncontrolled environments with non-laboratory tools may not always be highly accurate (Fuller et al., 2020). If, in fact, feedback alone—regardless of its accuracy—affects emotional experience simply by increasing self-directed attention, then the precision of physiological measurement becomes less critical. In this regard, studies on the effects of fake physiological feedback on emotion perception are also particularly relevant. These studies have shown that presenting entirely false (i.e. inaccurate) feedback on one’s physiological reactions, such as fabricated heart rate data, can lead individuals to perceive emotions (e.g. fear or romantic attraction) more intensely (e.g. Valins, 1966).

A different (yet interrelated) line of research showed that emotional significance usually improves the retention of events in long-term memory (LaBar & Cabeza, 2006). On the whole, events accompanied by emotions tend to be remembered better than events lacking an emotional component (cf., e.g. Kensinger & Kark, 2024; Kensinger & Schacter, 2008). Numerous studies have demonstrated that either an enhancement of stimulus arousal or a change in its valence (i.e. a shift from neutral in either direction, positive or negative) is sufficient to boost memory for that stimulus (Kensinger & Corkin, 2003). Crucially, even when there appear to be no differences between emotional and non-emotional (neutral) events in terms of accuracy in recognizing that the event occurred, differences emerge when considering the quality of the memory (i.e. the vividness and richness of details with which the event is remembered).

Such an emotional boost is often comparable for positive and negative stimuli (Kensinger & Kark, 2024), but in some cases, it has been found to be larger for negative events (e.g. Dewhurst & Parry, 2000; Ochsner, 2000), while in other cases, it is larger for positive ones (D’Argembeau et al., 2003; Schaefer & Philippot, 2005). These discrepancies might be attributed to the type of memory required by the task: there is evidence suggesting that positive emotions enhance the ability to remember the “gist” (general semantic theme) of an event, while negative emotions might enhance memory for specific details (Kensinger & Kark, 2024). These discrepancies might also depend on the information that participants consider most relevant to their goals. When stimuli associated with positive and negative emotions are equally relevant to one’s current concerns, they seem to be processed and remembered equally well (Kensinger & Schacter, 2008).

The fact that emotional stimuli are remembered better is not surprising: memories are believed to be stored as a collection of attributes, which may include emotional aspects (cf., Buchanan, 2007), and the richer a memory trace, the better its retention and retrieval (cf., Hargreaves et al., 2012). Therefore, one may hypothesize that the more people are aware of their emotions while experiencing events, the richer the related memory traces, and the better people can remember these events.

Based on these premises, one should expect that museum visitors wearing devices capable of measuring physiological responses to emotional events would process these responses more deeply and have a better awareness of them, even if they are not provided with real-time feedback about such responses. Consequently, these visitors might experience more vivid or intense emotions during the visit: the change in either the emotional valence or arousal induced by the visit experience might be enhanced or simply be more clearly detected. Moreover, since emotional events are thought to be better retained in long-term memory than non-emotional ones (LaBar & Cabeza, 2006), experiencing more vivid, distinct or intense emotions might also result in enhanced recollection of the museum visit. If this scenario holds true, then the use of physiological measurement devices would improve both the memorability and either the intensity or vividness/distinctness of the emotional experience during the visit, ultimately contributing to a more positive overall experience.

In contrast, visitors could also perceive the measurement of physiological indices as a kind of intrusive “monitoring pressure”, that is, as the constant and unpleasant presence of an unknown experimenter during their museum visit. Indeed, visiting a museum is a different experience from participating in a laboratory experiment, where one expects to be observed and monitored by the experimenter to some extent. Monitoring physiological changes during the visit could prompt visitors to experience sensations akin to the feeling of being eerily watched. If this is the case, then wearing physiological monitoring devices may compromise the overall experience of the visit, as well as visitors’ attentional allocation abilities (Belletier et al., 2015). Consequently, the memory encoding of the artworks might be impaired by wearing these devices, rather than improved.

Finally, one must also consider that people usually struggle to consciously monitor their physiological states. The so-called biofeedback, for example, can bring consistent benefits to people’s physical (e.g. Giggins et al., 2013) and psychological (e.g. Frank et al., 2010) health by providing otherwise unknown physiological information in real time. Accordingly, even if museum visitors wearing physiological measurement devices might be prompted to self-direct their attention and self-monitor physiological changes during the visit, awareness of such changes might not rise without appropriate feedback. If this is the case, then the emotional content of mnemonic traces related to a museum visit cannot be heightened solely by informing visitors that physiological changes are being measured; some form of actual feedback might be necessary.

As noted above, there is evidence that the emotional experience (e.g. its intensity or valence) can be manipulated by providing visitors with feedback about physiological changes and that this can occur irrespective of whether or not such feedback corresponds to the actual physiological states of the visitor (i.e. whether the feedback is correct and accurate or fake; Crucian et al., 2000; Valins, 1966). During real-time biofeedback training, people receive immediate information about their physiological states and learn to rely on it to overcome distressing conditions (e.g. anxiety and stress; Goessl et al., 2017). Even delayed biofeedback (i.e. feedback about an individual’s physiological states provided with a time lag rather than in real-time) can be effective in addressing stress-related symptoms (Wickramasekera, 1986). Accordingly, museum visitors receiving information about the physiological states that occurred during the visit, either in real time or at the end of the visit, may rely on it to interpret or reinterpret the emotions they experienced. In other words, visitors’ perception of their physiological experience during the visit and the emotional encoding of artworks could be driven or altered by appropriate feedback. This, in turn, might also influence the mnemonic encoding of the artworks. Investigating these aspects holds important implications for both museum curators and psychology researchers, as it could help us understand the influence that the perception of one’s physiological states has on aesthetic emotions and the memory of artworks and consequently encourage (or discourage) the provision of feedback regarding one’s physiological responses during museum visits.

### The present study

The present study consists of an in situ experiment conducted within the context of an art museum exhibition. The main focus of this study is to investigate whether wearing a physiological measurement device triggered intrusive monitoring pressure or, instead, promoted a positive and memorable experience. In detail, we tested whether the use of a non-invasive heart rate measurement device (a wristband) during the museum visit led visitors to experience more pleasant emotions (i.e. pleasing aesthetic emotions, which may include positive excitement as well as relaxation; Schindler et al., 2017) and improve their memory of the visit. A secondary objective aimed to determine whether it was possible to influence the accuracy of visitors' memories of the artworks by providing them with (fake) feedback about their physiological states after completing the museum visit. To this end, regardless of the actual physiological responses experienced by participants, once the museum visit was over, we presented one of the rooms in the exhibit as the one in which physiological responses indicative of pleasant emotions had been experienced.

Visitors took a tour inside a temporary exhibition at the Museum of Modern and Contemporary Art (MART) in Rovereto (Italy) and then filled out a questionnaire on emotional and aesthetic perception (see the Method section for details). Then, they completed an unexpected artwork recognition memory task, and, after at least six days, they repeated the questionnaire and the artwork recognition memory task remotely.

## Method

### Design and rationale

A 2 (Group: experimental vs. control) X 2 (Time of the testing: immediate vs delayed) mixed factorial design was used in the experiment. Time was a within-subject factor as all participants were tested both immediately after the visit and after at least six days. Group was a between-subject factor: about half of the participants were assigned to the experimental group and wore the heart rate monitoring wristband for the whole duration of the museum visit, while the remaining participants were assigned to the control group and did not wear the wristband. Participants in the experimental group were informed that psychological measurements related to emotions were being recorded during the visit. They were also told they would receive feedback consistent with their emotional experience. Following the completion of the aesthetic emotions questionnaire, all experimental participants were provided with the same (fake) feedback about their physiological responses (i.e. a slower heart rate) and emotions (i.e. a feeling of calm) allegedly experienced in one specific room of the exhibition compared to the other rooms. We reasoned that the awareness of being monitored through the wristband and the expectation of receiving feedback related to this monitoring might prompt participants to delve more deeply into their emotional responses to the artwork during the visit. The feedback received after filling in the questionnaire might also prompt them to reconsider and further elaborate on their overall visit experience. Participants in the control group only received information about the emotions experienced by the majority of other visitors in one room. This general feedback should not induce participants to re-elaborate on their visit, but rethinking this room and assigning it emotional meaning might nonetheless affect the memory of the artworks in that specific room.

To assess the impact of (personal versus general) feedback on the memory of the specific room mentioned to participants after the visit, we compared the artwork recognition accuracy of experimental and control participants in this room (both immediately and after a delay) with that of another room containing the same number of artworks.

### Participants

Seventy participants (52 female), aged between 18 and 55 (mean age = 25.16, sd = 6.82) with normal or corrected to normal vision, received a free museum ticket for their participation. Participants were randomly assigned to the two groups: 31 participants (22 female) were assigned to the experimental group and 39 (30 female) to the control group. Some participants did not fully complete the second part of the experiment (i.e. the delayed session). Hence, we included in the analyses only participants who had completed both the artwork recognition task and the emotions questionnaire when presented for the second time: In total, 52 participants were included (experimental = 26, control = 26). The study was approved by the ethics committee of the University of Trento (protocol code: 2021–046).

### Materials

#### Museum exhibit

**Fig. 1:**
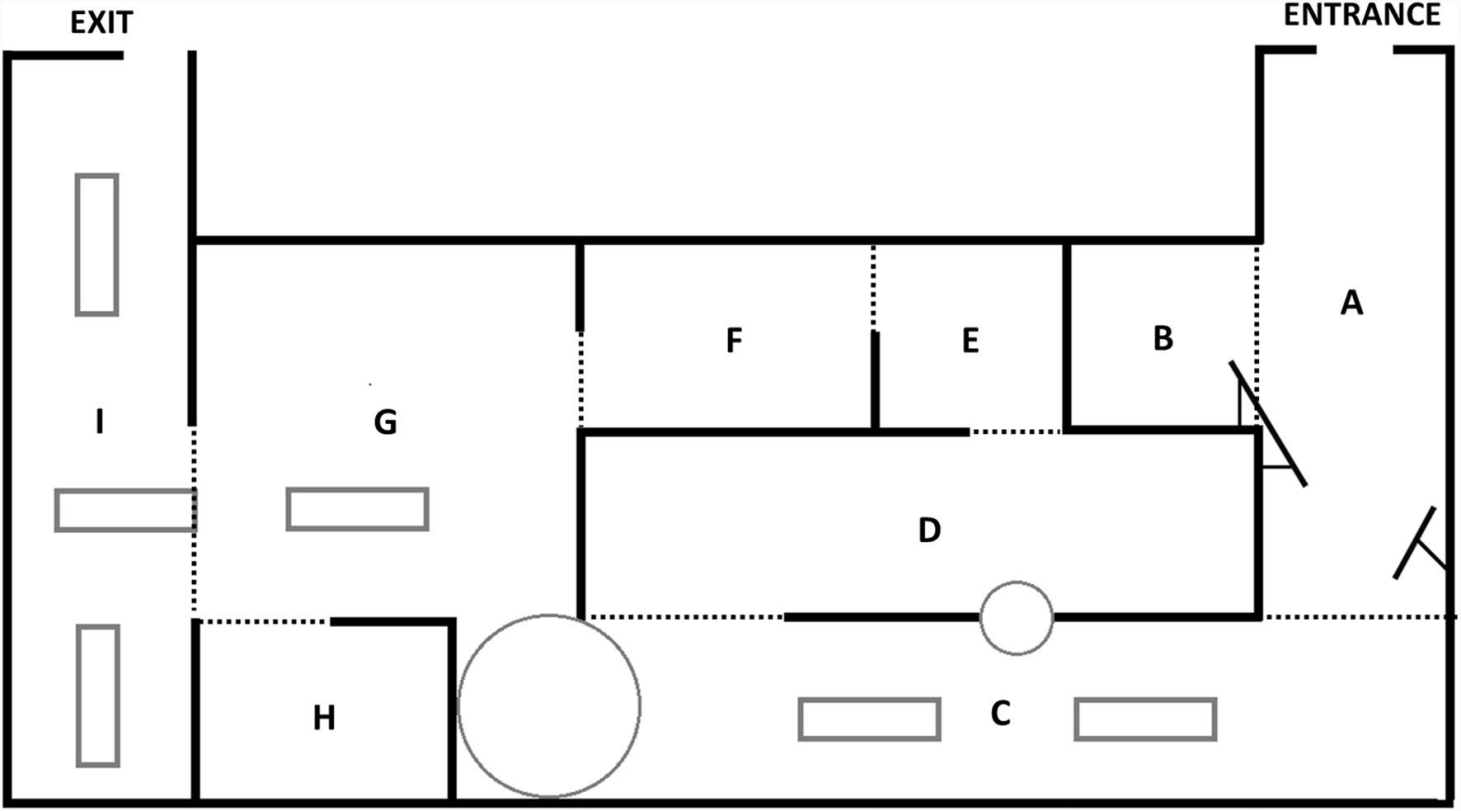
Stylized representation of the exhibition layout. The rooms are named using alphabetic letters, where A is the room closest to the entrance and I is the room closest to the exit. Shapes outlined in grey represent non-walkable art installations, while dotted lines mark the perimeter of the rooms

The visit took place in the context of the temporary exhibition “Depero New Depero”, which was displayed only from 21 October 2021, to 13 February 2022. We conducted the experiment in a temporary exhibition to ensure no one was familiar with the featured artworks. The exhibition consisted of 9 rooms of different sizes (see Fig. 1).

A total of 168 artworks by artist Fortunato Depero (1892–1960) or other artists associated with the Italian artistic movement known as “Futurismo” were displayed in the exhibition. For the purpose of this study, we chose to group the artworks into three macro-categories: paintings (42 in total) included colour artworks produced in cloth tarsia, fabric tarsia, tapestries, oil on canvas or tempera and watercolours on paper; sketches (76 in total) included black and white artworks produced in pencil, ink or charcoal on paper; finally, 3D objects (50 in total) included three-dimensional artworks, such as clothing, masks or wooden, glass, ceramic, tin, and metal structures (for a summary of the arrangement of artworks, see Table 1).

**Table 1:** Number and types of artwork pieces in each room of the exhibition. For each room, the number of pieces selected for the recognition task is also presented

Room	Paintings	Sketches	3D Objects	Total	Selected for the recognition task
A	2	1	1	4	3
B	2	0	2	4	3
C	8	31	14	53	18
D	3	12	1	16	10
E	1	0	16	17	8
F	3	0	1	4	6
G	9	15	13	37	8
H	5	13	0	18	10
I	9	4	2	15	6

#### Aesthetic emotions questionnaire

In order to assess the emotional response elicited by visiting the museum exhibit, we used a shortened version of the Aesthetic Emotions Scale (Aestemos; Schindler, et al., 2017).

The questionnaire was administered in Italian using the cloud-based software Qualtrics. It consisted of 14 sentences describing the emotional feelings a visitor might experience during a museum visit, grouped into 7 subscales: Feeling of beauty, Fascination, Enchantment, Vitality, Relaxation, Feeling of ugliness, and Boredom (see Table 2). The order in which the sentences were presented was random. Participants were asked to indicate how much each phrase reflected the emotional experience they felt during the visit on a scale from 1 (“Not at all”) to 4 (“Very much”).

**Table 2:** List of the sentences included in the aesthetic emotions questionnaire

Subscale	Item
Feeling of beauty	Liked it
	I found it beautiful
Fascination	Was impressed
	Fascinated me
Enchantment	Felt something wonderful
	Was enchanted
Vitality	Spurred me on
	Invigorated me
Relaxation	Calmed me
	Relaxed me
Feeling of ugliness	I found it distasteful
	I found it ugly
Boredom	Felt indifferent
	Bored me

#### Artwork recognition memory task

The artwork recognition task was designed drawing from the study of Ishai, et al. (2007) and was controlled by software developed in E-Prime (version 3.0.3.80). It was a typical old/new recognition task. During this task, a series of images of artistic works were presented to the participants, one at a time: the participants’ goal was to determine whether the image presented on the screen depicted an artwork displayed in the exhibit they had just visited.

Since the task was to be performed twice by each participant (i.e. immediately after the visit and at least six days after the visit), we devised two variants of the task. A different variant of the task was administered on the two occasions.

**Fig. 2:**
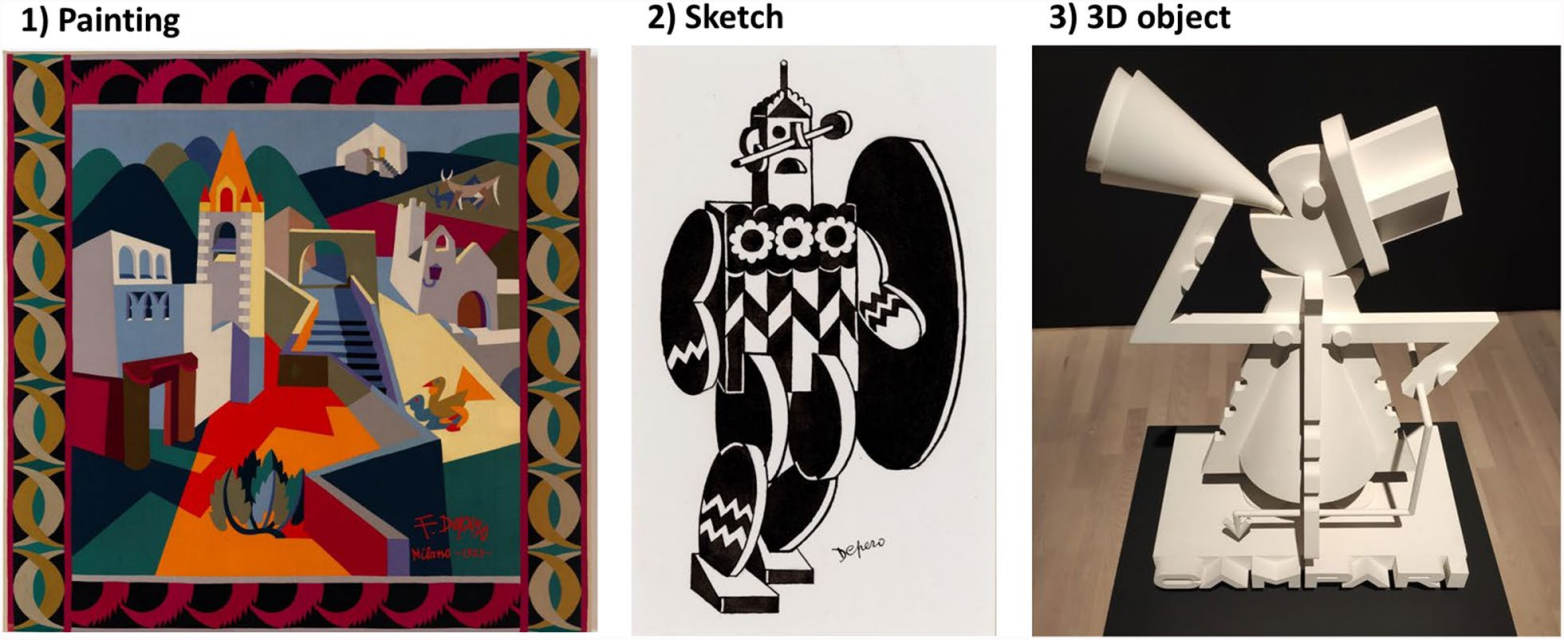
Examples of images depicting artworks displayed in the exhibition

Each variant was composed of 72 images depicting artworks that were present in the exhibition and 72 images depicting artworks that were not present. Of the 72 images depicting present artworks, 24 were of artworks displayed in the first three rooms (A, B, and C), 24 in the middle three rooms (D, E, and F), and 24 in the final three rooms (G, H, and I). In total, the images of present artworks depicted 16 sketches, 32 paintings, and 24 3D objects (see Fig. 2 for images’ examples). To prevent the images that were not present in the exhibition but were seen in the first-administered version of the recognition task from being mistakenly remembered as present in the second administration of the task, two groups of 72 images depicting artworks that were not present in the exhibition were selected. The version of the task administered immediately after the visit contained one of the two groups of images, while the version administered after the delay contained the other group. Each group of images depicting artworks not presented in the exhibition comprised the same number of sketches, paintings, and 3D objects as the group of images depicting artworks presented in the exhibition. In each group of non-present artworks, six “liar” (i.e. implausible) images depicted artworks that could not be present in the exhibition, as they belonged to artists outside the 'Futurismo' movement (e.g. Van Gogh’s “Starry Night”, the Statue of Liberty or Da Vinci’s “Vitruvian Man”). The remaining 66 images depicted artworks by artists associated with the “Futurismo” current or by Fortunato Depero himself, aligning with the theme of the exhibition.

In summary, the two variants of the artwork recognition task contained 72 images of artworks present in the exhibition (the same for both variants) and 72 non-present images (different for each variant). Each participant saw both variants. The order in which the two variants were presented was counterbalanced across participants so that each image depicting a non-present artwork was seen only once by any given participant.

In each trial, a fixation point was presented for 500 ms and was followed by the target image, which remained on the screen for 3 s and then disappeared. The sequence of image presentation was determined randomly. Participants were instructed to respond quickly, before the image disappeared, by pressing either a left or a right key on the keyboard (“D” or “L” characters, respectively, marked with tape) to indicate whether or not the image depicted an artwork displayed in the exhibition (e.g. a left keypress meant “Yes, I have seen it at the exhibition”, while a right keypress meant “No, I have not seen it at the exhibition”, or vice versa). Participants could still respond even once the image had disappeared, for an unlimited time. The yes/no (i.e. old/new) meaning of the keys was displayed on the screen for the entire duration of the task. Response mapping was counterbalanced across participants. The intertrial interval was set to 1 s.

### Procedure

Participants were guided by the experimenter in front of the entrance to the “Depero new Depero” exhibit. Participants in the experimental group were asked to wear an Amazfit Band 5 wristband, a commercially available device capable of heart rate monitoring. The wristband was described as follows, “*This is an experimental wristband, which allows us to measure your heart rate. The wristband will be active for the duration of the visit. Since the wristband is in the experimental stage, the measurements taken cannot be interpreted clinically in any way.*”. Participants belonging to the control group were not asked to wear the wristband.

All participants were invited to conduct a short visit, on their own, within the temporary exhibition. They were told that after the visit they would receive feedback consistent with (a) their emotional experiences of the visit (i.e. for the participants in the experimental group) or (b) emotional experiences of previous visitors of the exhibition. Participants were asked not to spend more than one hour inside the exhibition and not to revisit the rooms they had already explored. After the visit, the experimenter met participants at the exhibition’s exit and asked them to fill out the questionnaire on a tablet.

**Fig. 3:**
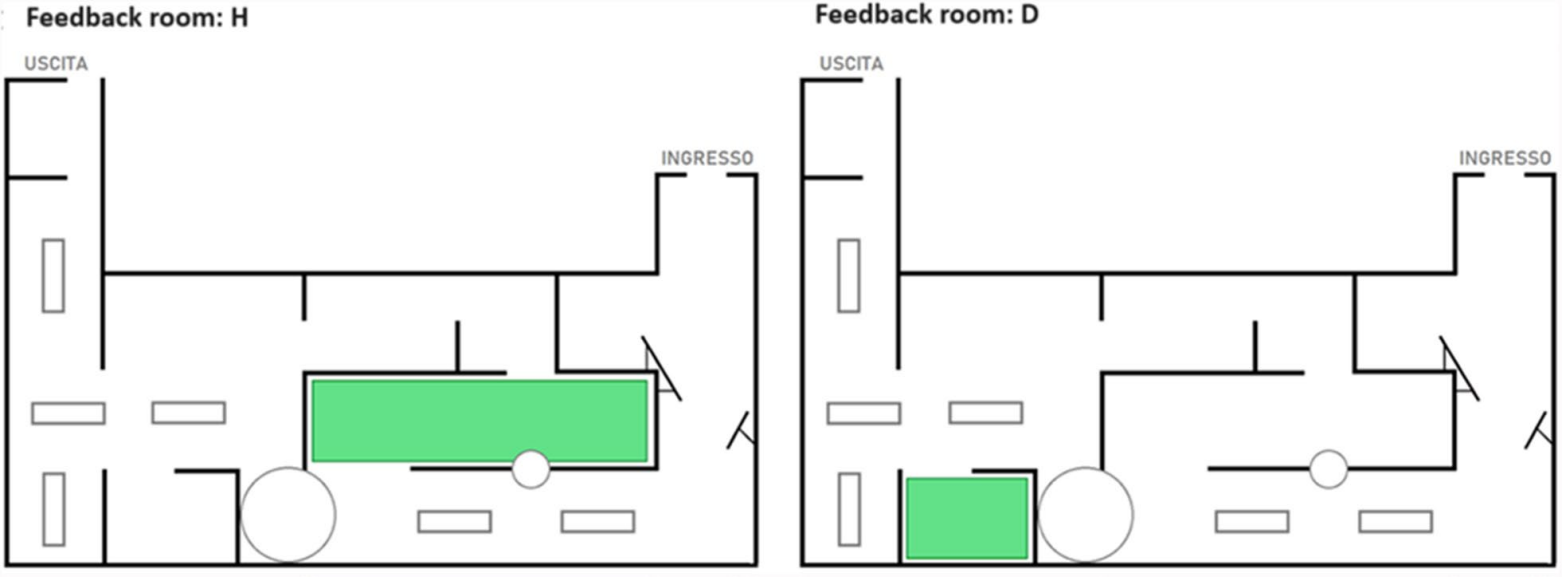
Feedback images given to participants. “INGRESSO” means entrance, while “USCITA” means exit

After completing the questionnaire, participants were invited to view an image created ad hoc for the experiment, depicting the floor plan of the exhibition with one room highlighted in green (see Fig. 3), and received (fictitious) feedback concerning the physiological states and emotions experienced in this room. For each participant, either the H or D room of the exhibition was chosen as the feedback room, while the other room served as the control room. These two rooms were indeed comparable as they contained approximately the same number of artwork pieces. Moreover, the same number of artworks were selected from each of these rooms for the recognition task (see Table 1). The H room was the feedback room for around half of the participants in each group, while the D room was the feedback room for the other half.

Participants in the experimental group received personal feedback, that is, feedback referring to their own emotional experiences. The highlighted room was described to them as follows: “*The room highlighted in green shows the room of the exhibition where your heart rate was slower. The paintings in this room are therefore the ones that had the most calming effect on you.*”. Participants in the control group received general feedback, that is, feedback referring to the physiological experience of other visitors who had made the same visit in the past. The highlighted room was described to them with these words: “*Within this floor plan, the room in which self-reported feelings of calm were recorded in a previous study was highlighted in green. This room is the room in which visitors usually feel calmest.*”.

Next, the examiner orally asked the participant two questions and marked the answers on a piece of paper: 1) “*Have you figured out which room it is?*” and 2) “*Do you relate to this description?*”.

After that, participants were escorted to the nearby laboratories of the Department of Psychology and Cognitive Science of the University of Trento, where they were unexpectedly asked to perform the artwork recognition task. Approximately 45 min elapsed from leaving the exhibition to the start of the recognition task.

Finally, six days after the visit, participants received an email asking them to perform a second version of the artwork recognition task (different from the version performed immediately after the visit, as described in the Materials section) and to fill out the emotions questionnaire again (the same version they had filled out before). Some participants did not complete both tests on the sixth day after the visit; as a result, the delayed session took place between 6 and 14 days after the visit (mean = 7.21 days, sd = 1.71). In this session, both the aesthetic emotions questionnaire and the artwork recognition task were performed remotely, the former through Qualtrics and the latter through E-Prime GO software.

## Results

Analyses were performed using the software RStudio (version 2022.02.0 + 443). The alpha level was set at 0.05, and partial eta-squared (*η*_***p***_^***2***^) is reported as the measure of effect size.

### Aesthetic emotions questionnaire

To assess the questionnaire’s reliability, we computed Cronbach’s alpha for the questionnaire items (Tavakol & Dennick, 2011): Alpha’s coefficients were sufficiently high for each item (i.e. between 0.83 and 0.87).

In order to evaluate the effect of wearing a wristband on self-reported emotions, we calculated the score for each subscale of the questionnaire by averaging participants’ responses across the two items composing the subscale (see Table 2).

**Fig. 4:**
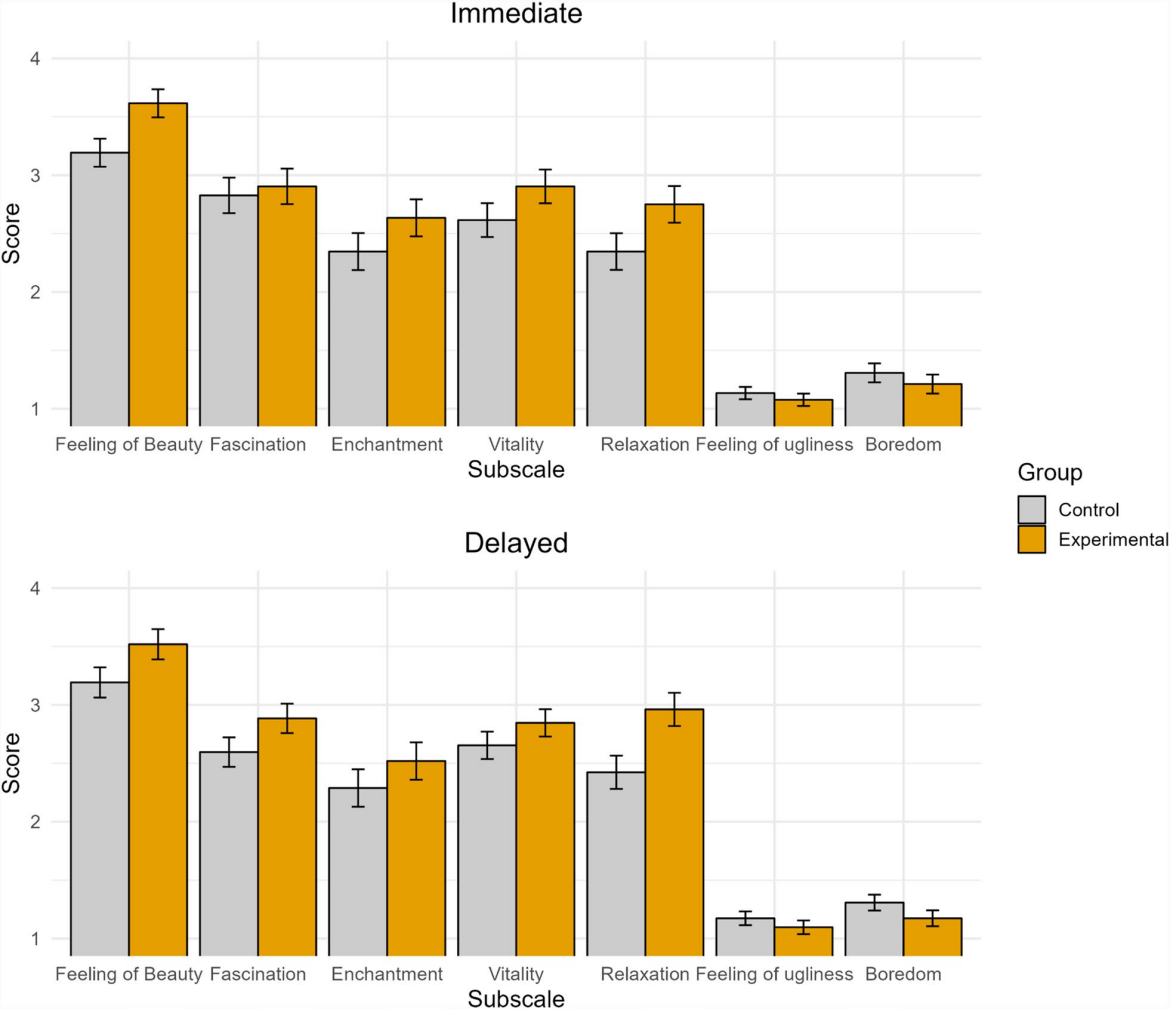
Mean scores measured in each subscale of the Aesthetic Emotions Questionnaire. The upper panel displays the first Time of testing condition (immediate), while the bottom panel displays the second Time of testing condition (delayed). In this figure, no items were reverse-coded. Vertical bars represent standard errors

Two Multivariate Analyses of Covariance (MANCOVA) were conducted with Group (experimental vs. control) as a between-subject factor, Time of testing (immediate vs. delayed) as a within-subject factor and Days elapsed between the two sessions as a covariate. The first MANCOVA included the five subscales associated with pleasant emotions as dependent variables (i.e. Feeling of beauty, Fascination, Enchantment, Vitality and Relaxation), while the second MANCOVA included the two subscales associated with unpleasant emotions as dependent variables (i.e. Feeling of ugliness and Boredom). A significant main effect of Group was observed only in the MANCOVA assessing subscales related to pleasant emotions, *F*(5, 45) = 2.51, *p* = 0.043, *η*_***p***_^***2***^ = 0.023. All the remaining effects, including the Group × Time of testing interaction, failed to reach significance (all *Fs* ≤ 1.04). This suggests that wearing a wristband influenced the participants’ self-reported pleasant emotions, and this effect did not change between the two times of testing (see Fig. 4).

To further explore the main effect of Group on the single subscales, mean scores of each subscale were entered as dependent variables in five different mixed-effect Analyses of Covariance (ANCOVA), with the same factors and covariate as those of the MANCOVAs. The main effect of Group was significant both when the Feeling of beauty subscale was tested, with the experimental group scoring higher than the control group, and when the Relaxation subscale was tested, with the experimental group showing higher scores than the control group, *F*(1, 49) = 5.25, *p* = 0.026, *η*_***p***_^***2***^ = 0.097, and *F*(1, 49) = 6.03, *p* = 0.018, *η*_***p***_^***2***^ = 0.110, respectively. All the remaining effects did not yield significant results (all *ps* > 0.05).

Overall, it seems that wearing a wristband that was thought to measure heart rate affected the participants’ self-reported responses associated with pleasant emotions. In particular, participants in the experimental group exhibited higher scores related to the feeling of beauty during the visit and reported being more relaxed (see Fig. 4).

### Artwork recognition memory task

**Fig. 5:**
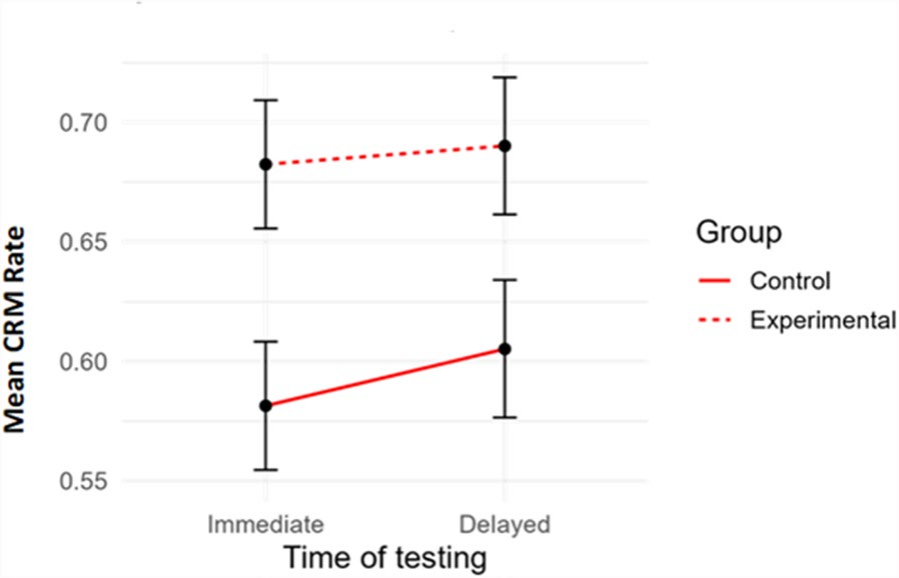
Mean Corrected Recognition Memory (CRM) rates, sorted by Time of the testing (immediate vs. delayed, on the x-axis) and Group (control vs. experimental). Vertical bars represent standard errors

In each version of the task, all participants identified at least five out of the six “liar” images (i.e. images depicting implausible artworks) as new images (i.e. not depicting artworks seen in the exhibit), with most (67 out of 70) correctly identifying all six images. Therefore, no participant was excluded based on the criterion of response plausibility. Corrected recognition memory (CRM) rate was calculated for each participant as the hit rate (i.e. the number of times the participant correctly recognized an image depicting an artwork from the exhibit as an old, previously seen item, divided by the total number of old items) minus the false alarms rate (i.e. the number of times the participant incorrectly identified a new item as old, divided by the total number of new items; cf., Thomas et al., 2006; Stark et al., 2019). CRM rates were entered as the dependent variable in a mixed-effects ANCOVA, with Group (experimental vs. control) as a between-subject factor, Time of the testing (immediate vs delayed) as a within-subject factor, and Days elapsed as a covariate. Only the main effect of Group proved significant, with participants belonging to the experimental group showing higher CRM rates than participants belonging to the control group, *F*(1, 49) = 6.19, *p* = 0.016, *η*_***p***_^***2***^ = 0.112. The main effect of Time of testing and the Group × Time of testing interaction were not significant. These results indicate that wearing the wristband improved the memory of the artwork pieces, regardless of whether participants were tested immediately after the visit or after a delay of six days or longer (see Fig. 5).

Finally, we analysed whether the feedback received by participants after the visit had an effect on their memory of the artwork pieces specifically seen in the room for which feedback was given, and whether the type of feedback (personal vs. general) can make any difference. Participants’ responses to the examiner’s questions related to the feedback are reported in Table 3.

**Table 3:** Distribution of participants’ answers (number of yes and no responses) to the questions concerning the description of the emotions allegedly experienced by themselves (in the case of experimental participants) or by previous visitors (in the case of control participants) in a specific room (i.e. the feedback room) of the exhibition

Group	Question	Answer	
		Yes	No
Experimental	Have you figured out which room it is?	29	2
	Do you relate to this description?	22	7
Control	Have you figured out which room it is?	37	2
	Do you relate to this description?	15	22

We calculated participants’ hit rates specifically for items in the feedback and control rooms. For each room, we divided the number of items correctly recognized as old (i.e. previously seen) in the recognition task by the total number of items depicting artworks from that specific room (i.e. 10 in both rooms; see Table 1).^2^ In these analyses, we included 55 participants (experimental group = 27, control group = 28): compared to the previous analyses, we included three additional participants who did not complete the emotion questionnaire in the delayed session but completed the artwork recognition task for both testing conditions (i.e. immediate and delayed).

**Fig. 6:**
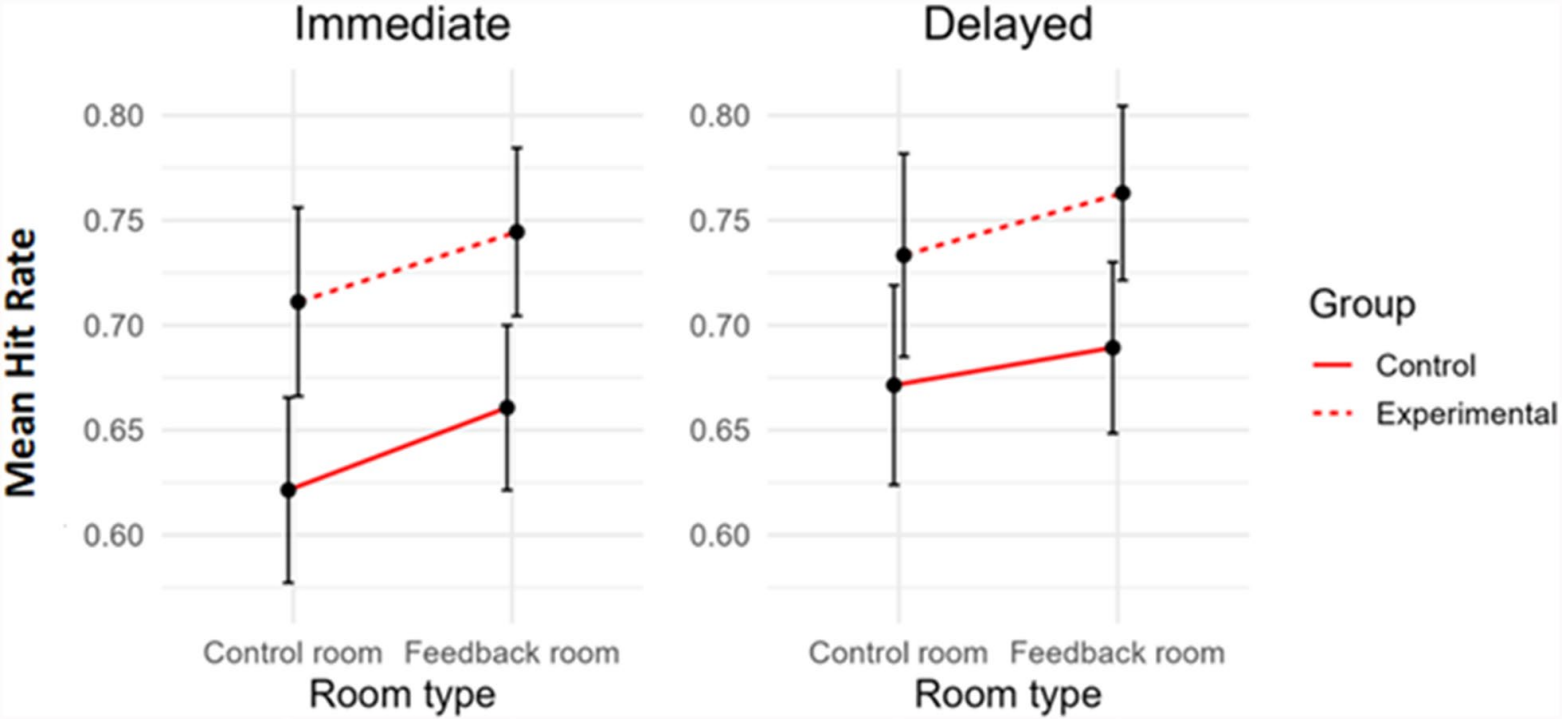
Mean hit rates in the two critical rooms of the exhibit, sorted by Room type (control room vs. feedback room), Group (control vs. experimental), and Time (immediate vs. delayed). Vertical bars represent standard errors

We performed a mixed-effect ANCOVA, with hit rates as the dependent variable, Room type (feedback room vs. control room) and Time of testing (immediate vs. delayed) as within-subject factors, Group (experimental vs. control) as a between-subject factor and Days elapsed as a covariate. Only the main effect of Room resulted significant, with artworks in the feedback room being better remembered than those in the control room, *F*(1, 52) = 4.10, *p* = 0.048, *η*_***p***_^***2***^ = 0.073. No other main effects or interactions were found to be significant (all *ps* > 0.05). These results suggest that the feedback improved participants’ memory of the artwork pieces, regardless of the Time of testing condition (see Fig. 6). Moreover, importantly, the factor Room type did not interact with the factor Group, *F* = 0.01, *p* = 0.92. There was no significant effect of Group either, *F*(1, 52) = 3.25, *p* = 0.077, even though the descriptive pattern mirrored that of the previous (general) analysis on CRM rates, with the experimental group outperforming the control group. Taken together, these results suggest a similar effect of the feedback in the two groups of participants: there were no substantial differences in the memory of the artworks seen in the feedback room between participants who received the personal feedback (experimental group) and participants who received the general feedback (control group).

In summary, these findings indicate that wearing a wristband that is designed to measure heart rate during a museum visit can improve memory of the artwork pieces for up to six days (or even longer). Moreover, receiving physiological and emotional feedback about a given room improves memory of the artwork pieces seen in that room, regardless of whether the feedback pertains to one's own emotional and psychological experience or to the general emotional and psychological states experienced by the majority of other people.

## Discussion

In this study, participants visited a temporary museum exhibition. The main objective was to investigate whether wearing a physiological measurement device could result in visitors experiencing more pleasant emotions and improve their memory of the visit. Interestingly, our findings provided evidence that wearing a wristband designed and believed to measure heart rate prompted visitors to report more pleasant emotions and improved their memory related to the artworks seen during the visit.

Specifically, when asked to fill out an aesthetic emotion questionnaire, participants wearing the wristband (i.e. the experimental group) reported higher ratings of pleasant aesthetic emotions compared to a control group, both immediately after the visit and even after a delay of at least six days. While scores suggesting a more pleasant emotional experience were obtained by experimental participants in all the subscales of the questionnaire (see Fig. 4), significant differences between groups in the self-reported intensity of emotions were only observed in the Feeling of beauty and the Relaxation subscales (i.e. experimental participants reported higher feelings of beauty and a greater sense of relaxation associated with the visit). This discrepancy between subscales could be caused by actual differences in the effect of our manipulation on different aesthetic emotions, but could also be attributed to the failure to detect genuine effects in some subscales due to the low sensitivity of the Likert rating system we used, which provided only 4 choices.

Additionally, when tested through an artwork recognition memory task, participants in the experimental group correctly recognized more artworks as belonging (or not belonging) to the exhibition they had visited than the control group, in both immediate and delayed sessions.

In line with the hypothesis presented in the Introduction section, these data suggest that wearing a device capable of measuring physiological responses to emotional events (i.e. being monitored through the wristband), and the expectation of receiving feedback related to this monitoring, increased the occurrence of pleasant emotions in participants visiting a museum. Based on the available evidence (e.g. Scheier et al., 1979), it is reasonable to assume that our manipulation increased the tendency to self-direct attention and prompted participants to delve into their emotional responses more thoroughly during the visit, making them more aware of emotions evoked by the artwork. As a consequence, either an increased likelihood of experiencing specific psychophysiological changes (whether arousing or relaxing) that are typically perceived as pleasant, an increased tendency to label them as pleasant, or an increase in the awareness of these changes lead experimental participants to experience more pleasant emotions (e.g. Kassam & Mendes, 2013; Pollatos & Schandry, 2008; Wiens, et al., 2000). This, in turn, may have triggered the cascade of events previously described, enriching the emotional component of participants’ memory traces of the artworks seen during the visit, improving visitors’ long-term encoding of these artworks and, ultimately, enhancing their memory of the visit (cf., LaBar & Cabeza, 2006; Talmi, 2013; Hamann, 2001; see also Hargreaves et al., 2012).

Nevertheless, it is important to acknowledge that this might not be the only explanation. One possible, different explanation of these findings is that the novel and extraordinary nature of the wristband (beyond and regardless of its physiological-monitoring objective) was sufficient to induce positive emotional changes (or better awareness of them) during the visit and, as a consequence, richer and better memories of the artworks. Alternatively, we may also propose that the more pleasant emotional experiences of the artworks and their improved memory encoding are due to physiological changes triggered by simply mentioning the heart rate measurement at the beginning of the visit. Indeed, informing the participants about the wristband’s heart rate measurement may have made physiological sensations more salient. These alternative accounts could be investigated in a future study by comparing memory and self-reported measurements of emotion between participants wearing a physiological-monitoring device during a museum visit, participants wearing a new and mysterious device not intended for any specific measurement, and participants not wearing any deceive but merely informed about measurement of physiological indices.

If the effects we observed in the present study are specifically due to wearing a device capable of measuring physiological responses and the expectation of receiving feedback from it—thanks to the increase in self-directed attention, the resulting enhancement of pleasant emotions, and the consequent improvement in the memory of the experience—then, in this future study, participants wearing a physiological monitoring device should express more positive emotional judgments and have a better memory of the experience compared to participants in the other two conditions.

If, instead, simply wearing an unusual device (regardless of its function) makes the experience more exciting and enhances both emotional experience and memory, then we should observe similar results in both conditions where participants wear a device. In both cases, we would expect more positive emotional judgments and better memory than in the condition where no device is worn.

Finally, if merely being aware that one’s physiological changes are being measured increases self-directed attention and influences emotional experience and memory, then we should observe similar results in both the condition where participants wear a physiological monitoring device and the condition where no device is worn, but participants are informed that some physiological measurement will take place. In both cases, we would expect more positive emotional evaluations and better memory than in the condition where participants wear a device unrelated to physiological measurement.

The results of this future study could therefore help to disentangle these three different accounts of the observed effects. It is important to note, though, that all these explanations involve a causal link between the increase in pleasant emotions reported by the experimental participants (regardless of its source) and their better performance on the memory test. This link was a key assumption of this study’s main hypothesis and a key element of the rationale behind our manipulation. However, it is also possible to hypothesize that the two findings are unrelated. Wearing the wristband may have led to the experience of more pleasant emotions, but this might not be the reason for the better performance on the recognition test. For example, we could assume that wearing the wristband increased experimental participants' motivation to engage more attentively with the exhibit, which, in turn, may have benefited their memory.

Alternatively, we could propose that the personal feedback given to the experimental participants (i.e. feedback about their own physiological and emotional experiences in one specific room compared to the other rooms of the exhibit) motivated them to thoroughly reconsider the content of the entire visit. This may have involved reflecting on the thoughts and emotions experienced in the various rooms of the exhibit and reconciling their personal experiences with the reported information. Since the feedback was provided after participants completed the emotion questionnaire, we can dismiss any impact of such reconsiderations on the self-reported emotional experience during the visit. However, we cannot rule out the possibility that this reconsideration may have contributed to the improved performance of experimental participants in the memory test. Indeed, a similar reconsideration of the entire museum visit may not be prompted by the general feedback given to the control participants. Notably, when asking participants if they related to the feedback description provided to them, only 15 out of 37 (41%) reported relating to the description in the control group, while the proportion was considerably higher in the experimental group, with 22 out of 29 (76%) reporting to relate with it (see Table 3). This suggests that fake feedback was somehow experienced differently based on its personal or general meaning.

However, it is also important to note that we did not observe an effect of the type of feedback (personal vs. general) on the memory of the artworks seen in the room for which participants received feedback. This was suggested by the absence of a significant interaction between Room type and Group (*F* = 0.01; see Results section). Indeed, a secondary objective of this study was to investigate whether it was possible to manipulate the accuracy of visitors’ memory of a specific room through (fake) feedback. We found that providing fake feedback portraying a room as the one in which participants experienced more pleasant emotions (i.e. “the room where you felt most relaxed”) enhanced the memory of the artworks in that room. Yet, this effect was observed for both experimental participants who received feedback regarding their own physiological responses and control participants who received generic feedback, involving the average physiological responses of other visitors. In other words, we showed that presenting fake feedback can influence visitors’ memory, independently of its personal or generic meaning. Crucially, this outcome also indicates that museum curators could implement physiological measurement devices without being overly concerned about their accuracy, as visitors would likely have a positive experience regardless.

Notably, the present study has some limitations. First, a considerable number of participants did not complete both sessions (*n* = 18), necessitating the exclusion of their data from the analysis. Moreover, the temporary art exhibition was relatively small and did not offer the flexibility to manipulate the presentation order of the artworks, which were shown in the same sequence to all participants. These limitations are inherent to the characteristics of temporary exhibitions, which are typically of short duration (making it challenging to gather a large number of participants) and are not amenable to modifications. In this study, as opposed to laboratory experiments where these variables can be meticulously controlled, we opted for an ecological context that aligns more closely with real-world scenarios. Finally, due to the limited number of artworks in the exhibition, we needed to use the same target artwork images in both the immediate and delayed versions of the recognition task. This might explain why the performance on this test was roughly the same in the two testing sessions and did not worsen from the immediate to the delayed session, as one might have expected. Testing the memory of the artworks from the exhibition in the immediate session and/or simply reviewing images of these artworks in the test administered during this session may have contributed to strengthening the memory of these artworks (cf., Roediger III & Karpicke, 2006) and explain the good performance in the delayed memory test. In the delayed test, participants may have had a sense of recognition and familiarity towards the target artworks both because they had seen these artworks in the exhibition and because they had viewed them again in the immediate test. However, it is worth noting that this did not overshadow the effect of our experimental manipulation (i.e. the effect produced by wearing the wristband during the exhibition): in the delayed test, experimental participants continued to outperform control participants.

## Conclusions

This study showed that visitors’ experience and memory of museum exhibitions can be improved when their physiological responses are recorded through appropriate devices. This may not be due to receiving accurate feedback (i.e. knowing which artworks elicited physiological response indicative of pleasant emotions), but rather simply result from wearing devices capable of measuring physiological activations. This holds significant implications for researchers and museum curators, as they can leverage such measurement devices to explore visitors’ physiological responses and enhance the overall enjoyment/memory of art exhibitions. Although further studies are required to draw stronger conclusions, we definitely view the result of the present study as a promising initial step. The results of this study also emphasize the need to be cautious in drawing conclusions from studies showing how real-time feedback to museum visitors can enhance their museum experience. The visitor's improved experience may not be due to knowing which artworks elicited certain emotional responses, but rather simply result from wearing devices capable of measuring physiological activations. Indeed, this awareness alone could bring visitors into closer contact with their emotional experiences during the visit.

## Data Availability

Data have been made publicly available at the Open Science Framework and can be accessed at https://osf.io/dqv6g/. The experiment was not preregistered.

## Footnotes

1 Some studies have explored the hypothesis that different emotional valences might be differentiated by physiological patterns involving a combination of indices rather than a single one (cf., e.g. Fruet et al., 2024).

2 False alarm rate could not be calculated for a specific room, as new images—not depicting artworks from the exhibition—did not belong to any room. As a result, it was not possible to use CRM rates for this analysis.

## References

[CR1] American Alliance of Museums (2023). Museum Visitation: A 2023 Annual Survey of Museum-Goers Data Story. https://www.aam-us.org/2023/11/10/museum-visitation-a-2023-annual-survey-of-museum-goers-data-story/#HTML.

[CR2] Barrett, L. F., & Russell, J. A. (1999). The structure of current affect: Controversies and emerging consensus. *Current Directions in Psychological Science,**8*(1), 10–14.

[CR3] Belletier, C., Davranche, K., Tellier, I. S., Dumas, F., Vidal, F., Hasbroucq, T., & Huguet, P. (2015). Choking under monitoring pressure: Being watched by the experimenter reduces executive attention. *Psychonomic Bulletin & Review,**22*, 1410–1416. 25673216 10.3758/s13423-015-0804-9

[CR4] Britton, J. C., Taylor, S. F., Berridge, K. C., Mikels, J. A., & Liberzon, I. (2006). Differential subjective and psychophysiological responses to socially and nonsocially generated emotional stimuli. *Emotion,**6*(1), 150–1555. 10.1037/1528-3542.6.1.150 16637758 10.1037/1528-3542.6.1.150

[CR5] Bruin, J., Stuldreher, I. V., Perone, P., Hogenelst, K., Naber, M., Kamphuis, W., & Brouwer, A.-M. (2024). Detection of arousal and valence from facial expressions and physiological responses evoked by different types of stressors. *Frontiers in Neuroergonomics.,**5*, 1338243. 10.3389/fnrgo.2024.1338243 38559665 10.3389/fnrgo.2024.1338243 PMC10978716

[CR6] Buchanan, T. W. (2007). Retrieval of emotional memories. *Psychological bulletin*, 133(5), 761. 10.1037/0033-2909.133.5.761 PMC2265099 17723029

[CR7] Carver, C. S., & Scheier, M. F. (1978). Self-focusing effects of dispositional self-consciousness, mirror presence, and audience presence. *Journal of Personality and Social Psychology,**36*(3), 324. 10.1037//0022-3514.36.11.1241 745034

[CR8] Ciolfi, L., & McLoughlin, M. (2012). Designing for meaningful visitor engagement at a living history museum. In Proceedings of the 7th nordic conference on human-computer interaction: Making sense through design (pp. 69–78).

[CR9] Citron, F. M., Gray, M. A., Critchley, H. D., Weekes, B. S., & Ferstl, E. C. (2014). Emotional valence and arousal affect reading in an interactive way: Neuroimaging evidence for an approach-withdrawal framework. *Neuropsychologia,**56*, 79–89. 24440410 10.1016/j.neuropsychologia.2014.01.002 PMC4098114

[CR10] Crucian, G. P., Hughes, J. D., Barrett, A. M., Williamson, D. J., Bauer, R. M., Bowers, D., & Heilman, K. M. (2000). Emotional and physiological responses to false feedback. *Cortex,**36*(5), 623–647. 10.1016/s0010-9452(08)70542-2 11195911 10.1016/s0010-9452(08)70542-2

[CR11] D’Argembeau, A., Comblain, C., & Van der Linden, M. (2003). Phenomenal characteristics of autobiographical memories for positive, negative, and neutral events. *Applied Cognitive Psychology: THe Official Journal of the Society for Applied Research in Memory and Cognition,**17*(3), 281–294.

[CR12] Dewhurst, S. A., & Parry, L. A. (2000). Emotionality, distinctiveness, and recollective experience. *European Journal of Cognitive Psychology,**12*(4), 541–551.

[CR13] Ekman, P., & Davidson, R. J. (Eds.). (1994). *The nature of emotion: Fundamental questions*. Oxford University Press.

[CR14] Ekman, P., Levenson, R. W., & Friesen, W. V. (1983). Autonomic nervous system activity distinguishes among emotions. *Science,**221*(4616), 1208–1210. 10.1126/science.6612338 6612338 10.1126/science.6612338

[CR15] Frank, D. L., Khorshid, L., Kiffer, J. F., Moravec, C. S., & McKee, M. G. (2010). Biofeedback in medicine: Who, when, why and how? *Mental Health in Family Medicine,**7*(2), 85. 22477926 PMC2939454

[CR16] Fruet, D., Mulatti, C., Treccani, B., Ferrante, D., & Nollo, G. (2024). Deep learning approach for response assessment to low intensity emotional stimuli. In IEEE 22nd MELECON, p. 508–513.

[CR17] Fuller, D., Colwell, E., Low, J., Orychock, K., Tobin, M. A., Simango, B., Buote, R., Van Heerden, D., Luan, H., Cullen, K., Slade, L., & Taylor, N. G. A. (2020). Reliability and validity of commercially available wearable devices for measuring steps, energy expenditure, and heart rate: Systematic review. *JMIR mHealth and uHealth,**8*(9), Article e18694. 32897239 10.2196/18694 PMC7509623

[CR18] Giggins, O. M., Persson, U. M., & Caulfield, B. (2013). Biofeedback in rehabilitation. *Journal of Neuroengineering and Rehabilitation,**10*, 1–11. 23777436 10.1186/1743-0003-10-60 PMC3687555

[CR19] Goessl, V. C., Curtiss, J. E., & Hofmann, S. G. (2017). The effect of heart rate variability biofeedback training on stress and anxiety: A meta-analysis. *Psychological Medicine,**47*(15), 2578–2586. 28478782 10.1017/S0033291717001003

[CR20] Google at 2019 Milan Design Week: A Space for Being. Youtube video, 3:27 (2019). *Posted by Google*. https://www.youtube.com/watch?v=4iA0srfIru0.

[CR21] Hamann, S. (2001). Cognitive and neural mechanisms of emotional memory. *Trends in Cognitive Sciences,**5*(9), 394–400. 11520704 10.1016/s1364-6613(00)01707-1

[CR22] Hargreaves, I. S., Pexman, P. M., Johnson, J. C., & Zdrazilova, L. (2012). Richer concepts are better remembered: Number of features effects in free recall. *Frontiers in Human Neuroscience,**6*, 73. 22514526 10.3389/fnhum.2012.00073 PMC3322485

[CR23] Haywood, N., & Cairns, P. (2006). Engagement with an interactive museum exhibit. In People and computers XIX—The bigger picture: Proceedings of HCI 2005 (pp. 113–129). Springer.

[CR24] Ishai, A., Fairhall, S. L., & Pepperell, R. (2007). Perception, memory and aesthetics of indeterminate art. *Brain Research Bulletin,**73*(4–6), 319–324. 17562398 10.1016/j.brainresbull.2007.04.009

[CR25] Kaplan, S., Bardwell, L. V., & Slakter, D. B. (1993). The restorative experience as a museum benefit. *Journal of Museum Education,**18*(3), 15–18.

[CR26] Kassam, K. S., & Mendes, W. B. (2013). The effects of measuring emotion: Physiological reactions to emotional situations depend on whether someone is asking. *PLoS ONE,**8*(6), Article e64959. 23785407 10.1371/journal.pone.0064959 PMC3680163

[CR27] Kensinger, E. A., & Corkin, S. (2003). Memory enhancement for emotional words: Are emotional words more vividly remembered than neutral words?. *Memory & cognition*, 31(8), 1169-1180. 10.3758/bf03195800 15058678

[CR28] Kensinger, E. A., & Kark, S. M. (2024). Emotion and memory. In E. A. Phelps & L. Davachi (Eds.), *Stevens’ handbook of Experimental Psychology and Cognitive Neuroscience* (Vol. 1, pp. 1–26). Wiley.

[CR29] Kensinger, E. A., & Schacter, D. L. (2008). Memory and emotion. In L. F. Barrett (Ed.), *Lewis M, Jones H-JM* (pp. 601–617). Guilford Press.

[CR30] Kissin, B. (2012). *Conscious and unconscious programs in the brain* (Vol. 1). Springer Science & Business Media.

[CR31] Kuppens, P., Tuerlinckx, F., Russell, J. A., & Barrett, L. F. (2013). The relation between valence and arousal in subjective experience. *Psychological Bulletin,**139*(4), 917–940. 10.1037/a0030811 23231533 10.1037/a0030811

[CR32] LaBar, K. S., & Cabeza, R. (2006). Cognitive neuroscience of emotional memory. *Nature Reviews Neuroscience,**7*(1), 54–64. 16371950 10.1038/nrn1825

[CR33] Larkin, K. T., Ciano-Federoff, L. M., & Hammel, D. (1998). Effects of gender of observer and fear of negative evaluation on cardiovascular reactivity to mental stress in college men. *International Journal of Psychophysiology,**29*(3), 311–318. 10.1016/S0167-8760(98)00019-1 9666384 10.1016/s0167-8760(98)00019-1

[CR34] Levenson, R. W., Ekman, P., & Friesen, W. V. (1990). Voluntary facial action generates emotion-specific autonomic nervous system activity. *Psychophysiology,**27*(4), 363–384. 2236440 10.1111/j.1469-8986.1990.tb02330.x

[CR35] Liu, X., Gurung, A., Baker, R. S., spsampsps Barany, A. (2024). Understanding the impact of observer effects on student affect. In Advances in Quantitative Ethnography, (pp. 79–94).

[CR36] Magsamen, S., & Ross, I. (2023). *Your brain on art: How the arts transform us*. Random House.

[CR37] Ochsner, K. N. (2000). Are affective events richly recollected or simply familiar? The experience and process of recognizing feelings past. *Journal of Experimental Psychology: General,**129*(2), 242. 10868336 10.1037//0096-3445.129.2.242

[CR38] Packer, J. (2008). Beyond learning: Exploring visitors’ perceptions of the value and benefits of museum experiences. *Curator: the Museum Journal,**51*(1), 33–54.

[CR39] Pollatos, O., & Schandry, R. (2008). Emotional processing and emotional memory are modulated by interoceptive awareness. *Cognition & Emotion,**22*(2), 272–287.

[CR40] Rickard, N. S. (2004). Intense emotional responses to music: A test of the physiological arousal hypothesis. *Psychology of Music,**32*(4), 371–388.

[CR41] Roediger, H. L., III., & Karpicke, J. D. (2006). Test-enhanced learning: Taking memory tests improves long-term retention. *Psychological Science,**17*(3), 249–255. 16507066 10.1111/j.1467-9280.2006.01693.x

[CR42] Schachter, S., & Singer, J. (1962). Cognitive, social, and physiological determinants of emotional state. *Psychological Review,**69*(5), 379. 14497895 10.1037/h0046234

[CR43] Schaefer, A., & Philippot, P. (2005). Selective effects of emotion on the phenomenal characteristics of autobiographical memories. *Memory,**13*(2), 148–160. 15847227 10.1080/09658210344000648

[CR44] Scheier, M. F., & Carver, C. S. (1977). Self-focused attention and the experience of emotion: Attraction, repulsion, elation, and depression. *Journal of Personality and Social Psychology,**35*(9), 625. 909041 10.1037//0022-3514.35.9.625

[CR45] Scheier, M. F., Carver, C. S., & Gibbons, F. X. (1979). Self-directed attention, awareness of bodily states, and suggestibility. *Journal of Personality and Social Psychology,**37*, 1576–1588. 10.1037/0022-3514.37.9.1576 501522 10.1037//0022-3514.37.9.1576

[CR46] Schindler, I., Hosoya, G., Menninghaus, W., Beermann, U., Wagner, V., Eid, M., & Scherer, K. R. (2017). Measuring aesthetic emotions: A review of the literature and a new assessment tool. *PLoS ONE,**12*(6), Article e0178899. 28582467 10.1371/journal.pone.0178899 PMC5459466

[CR47] Stark, S. M., Kirwan, C. B., & Stark, C. E. (2019). Mnemonic similarity task: A tool for assessing hippocampal integrity. *Trends in Cognitive Sciences,**23*(11), 938–951. 31597601 10.1016/j.tics.2019.08.003 PMC6991464

[CR48] Talmi, D. (2013). Enhanced emotional memory: Cognitive and neural mechanisms. *Current Directions in Psychological Science,**22*(6), 430–436.

[CR49] Tavakol, M., & Dennick, R. (2011). Making sense of Cronbach’s alpha. *International Journal of Medical Education,**2*, 53. 28029643 10.5116/ijme.4dfb.8dfd PMC4205511

[CR50] Thomas, R. C., & Hasher, L. (2006). The influence of emotional valence on age differences in early processing and memory. *Psychology and Aging,**21*(4), 821. 17201502 10.1037/0882-7974.21.4.821 PMC1764613

[CR51] Tschacher, W., Greenwood, S., Kirchberg, V., Wintzerith, S., van den Berg, K., & Tröndle, M. (2012). Physiological correlates of aesthetic perception of artworks in a museum. *Psychology of Aesthetics, Creativity, and the Arts,**6*(1), 96.

[CR52] Valins, S. (1966). Cognitive effects of false heart-rate feedback. *Journal of Personality and Social Psychology,**4*(4), 400–408. 10.1037/h0023791 5969996 10.1037/h0023791

[CR53] Wickramasekera, I. (1986). A model of people at high risk to develop chronic stress-related somatic symptoms: Some predictions. *Professional Psychology: Research and Practice,**17*(5), 437–447.

[CR54] Wiens, S., Mezzacappa, E. S., & Katkin, E. S. (2000). Heartbeat detection and the experience of emotions. *Cognition & Emotion,**14*(3), 417–427.

